# Bicalutamide Anticancer Activity Enhancement by Formulation of Soluble Inclusion Complexes with Cyclodextrins

**DOI:** 10.3390/biom12111716

**Published:** 2022-11-19

**Authors:** Federica De Gaetano, Maria Chiara Cristiano, Donatella Paolino, Consuelo Celesti, Daniela Iannazzo, Venerando Pistarà, Nunzio Iraci, Cinzia Anna Ventura

**Affiliations:** 1 Department of Chemical, Biological, Pharmaceutical and Environmental Sciences, University of Messina, Viale Ferdinando Stagno D’Alcontres 31, I-98166 Messina, Italy; 2 Department of Clinical and Experimental Medicine, University ‘Magna Græcia’ of Catanzaro, I-88100 Catanzaro, Italy; 3 Department of Engineering, University of Messina, Contrada Di Dio, I-98166 Messina, Italy; 4 Department of Clinical and Experimental Medicine, University of Messina, Via Consolare Valeria, I-98125 Messina, Italy; 5 Department of Pharmaceutical and Health Sciences, University of Catania, Viale Andrea Doria 6, I-95125 Catania, Italy

**Keywords:** bicalutamide, cyclodextrins, molecular modeling, in vitro biological studies, DU-145 and PC3 cell lines

## Abstract

Bicalutamide (BCL) is a nonsteroidal antiandrogen drug that represents an alternative to castration in the treatment of prostate cancer, due to its relatively long half-life and tolerable side effects. However, it possesses a very low water solubility that can affect its oral bioavailability. In this work, we developed inclusion complexes of BCL with the highly soluble hydroxypropyl-β-cyclodextrin (HP-β-CyD) and sulfobutylether-β-cyclodextrin (SBE-β-CyD) to increase the water solubility and anticancer activity of BCL. The inclusion complexes were prepared using the freeze-drying method and were then characterized in a solid state via differential scanning calorimetry and X-ray analysis and in solution via phase-solubility studies and UV-vis and NMR spectroscopy. The BCL/HP-β-CyD and BCL/SBE-β-CyD inclusion complexes were amorphous and rapidly dissolved in water. Both the ^1^H-NMR spectra and molecular modeling studies confirmed the penetration of the 2-(trifluoromethyl)benzonitrile ring of BCL within the cavity of both cyclodextrins (CyDs). Due to the consistent improvement of the water solubility of BCL, the inclusion complexes showed higher antiproliferative activity toward the human prostate androgen-independent cell lines, DU-145 and PC-3, with respect to free BCL. These results demonstrate the ability of HP-β-CyD and SBE-β-CyD to complex BCL, permitting the realization of liquid formulations with potentially high oral bioavailability and/or possible parenteral administration.

## 1. Introduction

Prostate cancer represents the second most common type of malignancy, after lung cancer, that affects men. It can be asymptomatic in the early stages; it often has an indolent course and may require only active surveillance. However, the most frequent complaints from sufferers are difficulty with urination, increased urination frequency, and nocturia related to prostatic hypertrophy. More advanced stages of the disease may present with urinary retention and back pain, probably due to bony metastatic disease [1]. The development of prostate cancer is attributable to a mutation in the androgen receptor (AR), which is fundamental in the genetic expression of many tissues, including the prostate [2]. AR regulates many cellular events, including apoptosis, differentiation, and cell proliferation. This makes its role relevant in prostatic oncological disease [3], which is classified in the first stage as a hormone-responsive disease. It is only later that it passes from an androgen-dependent to an androgen-independent phenotype and progresses from precursor lesions to a carcinoma, which is initially confined to the prostate but can lead to metastatic disease, often resulting in lethality [4].

The first-line treatment for prostate cancer, confined to this organ, is androgen deprivation therapy (ADT), specifically, surgical or pharmacological castration. However, after 2–3 years, many patients develop castrate-resistant prostate cancer (CRPC), for which treatment options are limited and the prognosis is poor [5]. Furthermore, in the longer term, castration, whether pharmacological or surgical, may increase the risk of osteoporosis [6], the reduction of cognitive function [7], and anemia [8]. ADT reduces about 90–95% of the serum circulating testosterone [9]; because this plays an important role in normal male sexual function, its decrease has a negative impact on the patient’s quality of life, producing a loss of libido and impotence [10]. Monotherapy with nonsteroidal antiandrogens, such as bicalutamide (BCL), is emerging as an attractive alternative to castration due to its relatively long half-life, which permits once-daily administration, and tolerable side effects. BCL does not suppress testosterone production and, therefore, offers potential quality-of-life advantages over castration. In advanced prostate cancer cases, BCL is used in combination with a gonadotropin-releasing hormone analog [11]. BCL exhibits a chiral center, and the two enantiomers possess different pharmacokinetics. The *R*-enantiomer (*R*-BCL) has 30 times higher affinity than the *S*-enantiomer (*S*-BCL) for androgen receptors in rat prostate tissue and it is responsible for most of the antiandrogenic activity of the drug [12]. BCL is well adsorbed after oral administration due to its high lipophilicity (log P, 2.92); however, it possesses very low water solubility (<40 mg/L) [13], which reduces its bioavailability (a class-II drug in the biopharmaceutical classification system). This drawback necessitates large doses in oral administration, reducing patient compliance. Furthermore, the poor water solubility of BCL prevents its formulation in a liquid form for parenteral administration.

Different strategies can be used to increase the water solubility of lipophilic drugs, such as solid dispersions [14], micellar solubilization [15], nanoemulsions [16], hydrotropic solubilization [17], particle size reduction [18], lipid-based delivery systems [19], and complexation with cyclodextrins (CyDs) [20,21]. Among them, CyDs represent an excellent opportunity to improve the biopharmaceutical properties of insoluble drugs [22]. For example, the inclusion of idebenone into hydroxypropyl-β-CyD (HP-β-CyD) was effective in improving its water solubility and dissolution rate [23] and prevent carrageenan-induced hyperalgesia and edema in a dose-dependent manner [24]. Native β-CyD and sulfobutylether-β-CyD (SBE-β-CyD) effectively include the bioflavonoid, coumestrol, enhancing its water solubility [25,26] and potentially improving its antioxidant and anti-inflammatory activity. Recently, the important role played by drug/CyD inclusion complexes was demonstrated, in terms of improving the encapsulation parameters and release rate of hydrophilic and hydrophobic drugs from polymeric nanoparticles [27,28], as well as the ability of these macrocycles to self-assemble into nanoaggregates, improving the water solubility of drugs and potentiating their therapeutic efficacy [29,30].

Recently, other authors have characterized the inclusion complexes of BCL with native β-CyD [31,32], HP-β-CyD [33,34], and methyl- and acetyl-β-CyD [35]. An increase in water solubility and the dissolution rate of BCL was observed, particularly when modified β-CyD was used as a complexing agent [34,35], due to its higher solubility compared to native β-CyD. Furthermore, the presence of substituents extends the size of the lipophilic cavity, improving its affinity for BCL. Even if the physical-chemical characterization of the BCL/CyD complexes was performed in these works, none of the papers explained, either in vitro or in vivo, the macrocycles’ influence on the anticancer activity of the included BCL.

**Figure 1:**
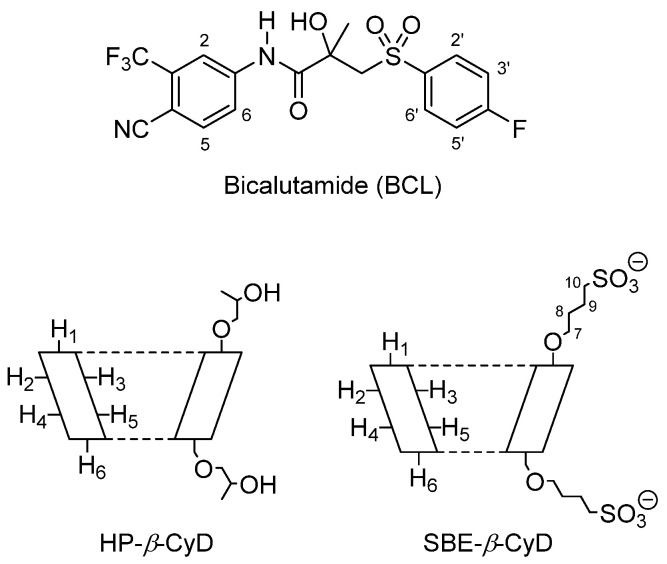
The molecular structure of BCL and the schematic structure of HP-β-CyD and SBE-β-CyD.

In this work, we developed highly soluble formulations based on the inclusion of complexes of BCL with HP-β-CyD and sulfobutylether-β-cyclodextrin (SBE-β-CyD) (Figure 1), in view of its potential use in prostate cancer treatment. Due to their safety, both HP-β-CyD and SBE-β-CyD were approved by the Food and Drug Administration (FDA) for parenteral administration [36]. Thus, their use can permit the realization of BCL formulations that are suitable for oral and/or parenteral administration. Soluble BCL/HP-β-CyD and BCL/SBE-β-CyD inclusion complexes were prepared using the freeze-drying method and were characterized in solution by UV-Vis spectroscopy and phase-solubility studies, to determine the stability constant (Kc) of the complexes. Differential scanning calorimetry (DSC) and X-ray analysis were used to characterize the inclusion complexes in a solid state. ^1^H-NMR spectroscopy and molecular modeling analyses were used to define the geometry of the complexes. Finally, for the first time, the in vitro anticancer effects of the complexes, in comparison with free BCL, were assayed on the human prostate androgen-independent cell lines, DU-145 and PC-3.

## 2. Materials and Methods

### 2.1. Materials

Bicalutamide (BCL) (C_18_H_14_F_4_N_2_O_4_S, molecular weight, 430.37) was purchased from Zentek SRL (Milano, Italy). First, 2-hydroxypropyl-β-cyclodextrin (HP-β-CyD, 0.6 molar substitution, average molecular weight, 1380 g/mol) was purchased from Sigma-Aldrich (St. Louis, MO, USA). Sulfobutyl-ether-β-cyclodextrin (SBE-β-CyD, CAPTISOL^®^; the average degree of sulfobutyl substitution is seven, with an average molecular weight of 2162 g/mol) was kindly supplied by CyDex Pharmaceutical (Lenexa, KS, USA). The water used throughout the study was double-distilled, then filtered through 0.22 μm Millipore^®^ GSWP filters (Bedford, MA, USA). All other products and reagents were of analytical grade.

### 2.2. Preparation of the Inclusion Complexes

The BCL/HP-β-CyD and BCL/SBE-β-CyD inclusion complexes were prepared, in 1:1 molar ratio, using the freeze-drying method. Briefly, HP-β-CyD (6.376 mg, 4.62 × 10^−6^ M) and SBE-β-CyD (10 mg, 4.62 × 10^−6^ M) were solubilized in water (8 mL), at room temperature, in stoppered flasks. After that, a methanol solution (2 mL) containing BCL (1.99 mg, 4.62 × 10^−6^ M) was added dropwise to each flask, under magnetic stirring. The obtained solutions were poured into freeze-drying flasks and placed into the vacuum chamber, frozen at −50 °C, and then freeze-dried for 72 h (VirtTis Benchtop K Instrument, SP Scientific, Warminster, PA, USA).

### 2.3. Differential Scanning Calorimetry (DSC)

DSC analyses of free BCL, HP-β-CyD, the inclusion complexes, and the physical mixtures were carried out using a TAQ500 instrument (TA Instruments, New Castle, DE, USA) under argon flow at a rate of 100 mL/min, heated from 25 °C to 350 °C, with a heating rate of 5 °C/min.

### 2.4. X-ray Analysis

All X-ray diffraction (XRD) experiments were performed at room temperature with a Bruker D8 Advance diffractometer (Bruker, Karlsruhe, Germany) using a Bragg–Brentano theta-2-theta configuration, with CuKa radiation (40 V, 40 mA). XRD patterns were collected in the range of 5–80°, with a step of 0.2°/s. The diffraction peaks were compared with those in the Joint Committee on Powdered Diffraction Standards (JCPDS) database.

### 2.5. Phase-Solubility Studies

Phosphate buffer solution (PBS, 10 mL pH 7.4), containing increasing amounts of HP-β-CyD or SBE-β-CyD (from 0 to 12 mM), was added to BCL in amounts exceeding its intrinsic solubility and then sonicated in a Bandelin RK 514 water bath (Berlin, Germany), for 15 min. The suspensions were placed in a thermostated bath (Telesystem 15.40, Thermo Scientific, Waltham, MA, USA), equipped with a temperature control unit (Telemodul 40C, Thermo Scientific, Waltham, MA, USA), at 25.0 ± 0.1 °C, under magnetic stirring for 7 days. After that, the suspensions were filtered through Sartorius Minisart-SRP 15-PTFE 0.22-µm filters (Bedford, MA, USA) and analyzed, via UV-vis spectroscopy, to evaluate the amount of solubilized BCL. Experiments were carried out in triplicate. The data obtained from the phase solubility diagrams were used to calculate the association constant (K_c_) of the inclusion complexes, according to the Equation (1):

(1) $$K_{c}=\frac{\mathrm{Slope}}{\left(1-\mathrm{Slope}\right)S_{0}}$$

where S_0_ is the intrinsic solubility of BCL [37].

### 2.6. Determination of Dissolution Rate

The dissolution rate of the complexes was determined in vitro, in accordance with the 44th United States Pharmacopoeia (USP) paddle method. Free or complexed BCL (5 mg) was suspended in 900 mL of PBS, at pH 6.8, and stirred at 100 rpm at 37.0 ± 0.5 °C. At fixed time intervals (5, 10, 15, 30, 45, 60, and 120 min), aliquots of the suspension were collected, filtered (Sartorius Minisart-SRP 15-PTFE, 0.22-µm filters, Bedford, MA, USA), and analyzed spectrophotometrically, to determine the amount of drug in the solution using the UV-vis apparatus described below. After each sampling, in order to maintain the sink conditions, the volume was adjusted to 900 mL with fresh preheated water. The experimental results were reported as an average of at least three replicated experiments.

### 2.7. UV-Vis Titration Studies

The UV-vis titration studies were performed as follows. Free BCL (4.65 × 10^−7^ M) or BCL, in the presence of increasing HP-β-CyD or SBE-β-CyD concentrations (BCL:CyD molar ratio, 1:1, 1:2, 1:5, 1:10, 1:20, 1:30, 1:50), was solubilized in water/methanol solution (60/40, *v*/*v*) and stirred for 24 h. The solutions were then analyzed via UV-vis spectroscopy, as reported below.

### 2.8. UV-Vis Spectroscopy

UV-vis spectral analysis was carried out in the spectral range of 200–400 nm, using a StellarNet BLACK-Comet, model C, diode array spectrophotometer (Florida, FL, USA), employing 1-centimeter rectangular quartz cells (Hellma, Milano, Italy). The calibration curve was prepared in water/methanol solution (60/40, *v*/*v*) at a λ_max_ of 272 nm, with concentrations ranging from 0.00032 mg/mL to 0.032 mg/mL. An R^2^ value of 0.9984 was obtained.

### 2.9. H-NMR Studies

Samples of equivalent concentrations (9 mM) of BCL, SBE-β-CyD, HP-β-CD, and the corresponding inclusion complexes were prepared in a D_2_O/CD_3_OD (8:2, *v*/*v*) solution and transferred to 5-mm NMR tubes for spectrum acquisition. All spectra were recorded at 300 K with a Varian Unity Inova 500 MHz (11.75 T) instrument. The deuterated methanol (3.30 ppm) was used as an internal reference, to avoid the addition of external references that could interact with the CyDs.

### 2.10. Molecular Modeling

#### 2.10.1. Preparation of Structures

The HP-β-CyD and SBE-β-CyD in silico models were built in a Maestro [38] GUI upon the crystal structure of β-cyclodextrin (CCDC entry 762697) [39]. HP-β-CyD was modeled according to a previously reported study, substituting the C2 oxygens of residues 1, 3, 5, and 7 with the 2-hydroxypropyl group [23]. SBE-β-CyD was modeled using substitutions of oxygens at C2 of residues 1, 4, and 7, at C3 of residues 3 and 6, and at C6 of residue 5, in accordance with the NMR data provided by the SBE-β-CyD manufacturer. The *R*-enantiomer structure of BCL was downloaded from PubChem [40] (ID 2375) and its chiral center was modified in Maestro to obtain the *S*-enantiomer.

#### 2.10.2. Molecular Dynamics

Two simulated environments were prepared by randomly placing the previously modeled molecules in two cubic periodic boundary conditions simulation boxes. The first system contained an HP-β-CyD molecule, an *R*-BCL molecule, and an *S*-BCL molecule, while the second one contained an SBE-β-CyD molecule, an *R*-BCL molecule, and an *S*-BCL molecule. Solvation was treated explicitly, using the TIP3P water model [41], and OPLS 2005 [42] was used as a force field. The systems were neutralized by Na^+^ and Cl^−^ ions, which were added until a 0.15 M concentration was reached. Before running the molecular dynamics (MD) of the production stages, all systems were relaxed by a previously reported multi-stage protocol [43]. Simulations were set up and run using Desmond [44].

To enhance the conformational sampling, MD simulations were run using the Replica exchange with the solute tempering (REST) method. [45]. Three replicas were set to 300 K, 341.25 K, and 385.72 K. The BCL molecules were set as hot regions. First, 480 ns-long simulations were run in the isothermal-isobaric NPT ensemble, with time steps set to 2 fs, 2 fs, and 6 fs for bonded, near, and far interactions, respectively. The structures were sampled every 120 ps and the trajectories were analyzed using visual molecular dynamics (VMD) [46]. MD snapshots (4000 frames) from replica 0 (t = 300 K) were clustered according to their atomic RMSD values, using complete linkage and 4 Å as the merge distance cutoff. Non-hydrogen ligand atoms and C1-O-C4 pattern atoms of HP-β-CyD or SBE-β-CyD were used as reference atoms for fitting and RMSD calculation.

### 2.11. Biological In Vitro Studies

#### 2.11.1. Culture Cells

The human prostate cells (DU-145 and PC-3) used in this study were kindly gifted by the University of Campania “Luigi Vanvitelli”, Naples, Italy. These cell lines were cultured in Dulbecco’s modified Eagle’s medium (DMEM), supplemented with 10% fetal bovine serum (FBS) and supplemented with 0.3 mg/mL l-glutamine, 100 IU/mL penicillin, and 100 μg/mL streptomycin. Both cell lines were cultivated in a 5% CO_2_-humidified atmosphere at 37 °C and subcultured using trypsin-EDTA when they were 85–90% confluent.

#### 2.11.2. In Vitro Evaluation of Cytotoxic Activity

The MTT assay (3-(4,5-dimethylthiazol-2-yl)-2,5-diphenyltetrazolium bromide, a yellow tetrazole) was performed to evaluate the cytotoxic activity of BCL, BCL/HP-β-CyD, and BCL/SBE-β-CyD inclusion complexes. DU-145 and PC-3 cells were seeded into 96-well cell culture plates (5 × 10^3^/0.66 cm^2^) and incubated at 37 °C. After 24 h of incubation, the cell culture medium was poured off and replaced with 0.1 mL/well containing the samples dissolved in PBS, at pH 7.4. Free BCL was assayed at 10 μM, 25 μM, 50 μM, 75 μM, and 100 μM concentrations. The inclusion complexes were assayed at 10 μM, 25 μM, 50 μM, and 75 μM concentrations, based on the included BCL. Untreated cells were used as a control during the experiments. The cytotoxic activity of compounds was evaluated at different incubation times (24 h, 48 h, and 72 h). An MTT solution (5 mg/mL in PBS, pH 7.4) was added to each well and further incubated for 3 h. The cell culture medium was then removed and the obtained formazan crystals, which had precipitated on the bottom of the well, were dissolved using 100 μL of a DMSO/ethanol (1/1, *v*/*v*) solution. The plates were further shaken for 20 min at 230 rpm (IKA^®^KS 130 Control, IKA^®^WERKE GMBH & Co., Staufen, Germany). The dissolved formazan crystals were quantified using a microplate spectrophotometer (BIORAD, MarkTM microplate spectrophotometer) at a wavelength of 570 nm by subtracting the background absorbance, measured at 690 nm. The percentage of cell viability was calculated according to the Equation (2):

(2) $$\mathrm{Cell}\;\mathrm{Viability}\;\left(\%\right)=\frac{\mathrm{AbsT}}{\mathrm{AbsC}}*100$$

where AbsT is the absorbance of treated cells and AbsC is the absorbance of control cells (untreated cells). Results are expressed as mean values of three independent experiments ± standard deviation.

#### 2.11.3. Statistical Analysis

All data are shown as the mean of the values obtained from the study, ± standard deviation. The statistical analysis comparing different groups was performed by a one-way ANOVA analysis with a Bonferroni post hoc test. A * *p*-value < 0.05 and a ** *p*-value < 0.001 were considered statistically significant.

## 3. Results and Discussion

### 3.1. Solid-State Characterization

**Figure 2:**
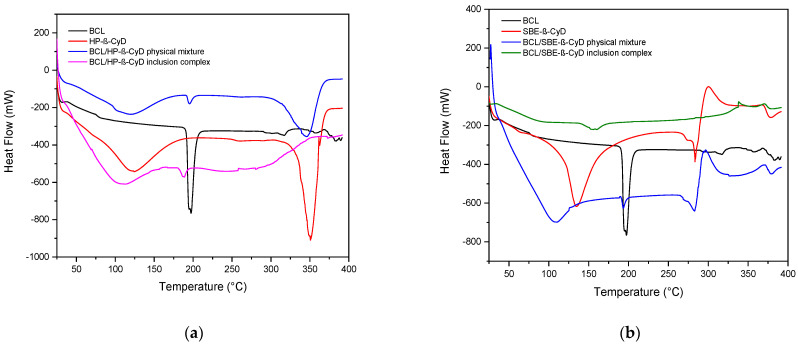
DSC thermograms of the BCL/HP-β-CyD (**a**) and BCL/SBE-β-CyD (**b**) systems. The experiments were performed under an argon atmosphere.

BCL/HP-β-CyD and BCL/SBE-β-CyD inclusion complexes were prepared using the freeze-dried method, starting from an aqueous/methanol solution. The organic solvent was needed to solubilize BCL and facilitate molecular complexation within the CyD cavities. The process produced amorphous powders, as demonstrated by DSC analysis and X-ray diffractograms. Figure 2a shows the thermograms obtained for the BCL/HP-β-CyD system. The inclusion complex was analyzed comparatively with the free drug, HP-β-CyD, and the physical mixture prepared to the same molar ratio as the inclusion complex. Free BCL shows a characteristic endothermic peak at 195 °C (ΔH 10.93 J/g), corresponding to the melting of the crystalline drug [13]. The thermogram of free HP-β-CyD presented two endothermic events, one at 121 °C, due to the loss of crystalline water, and the other one at 350 °C, corresponding to the macrocycle degradation. The BCL/HP-β-CyD physical mixture thermogram shows the superimposition of both free components. We could clearly observe the loss of water (at about 121 °C), the melting of free BCL (at 193 °C), and the degradation of the free macrocycle. A different trend was observed for the BCL/HP-β-CyD inclusion complex. In particular, the BCL melting peak was shifted toward a lower temperature (183 °C), and the degradation peak of the macrocycle was absent; it was probably shifted toward a higher temperature with respect to free HP-β-CyD and the physical mixture. This could demonstrate the presence of a new solid phase in which a solid-solid interaction between BCL and the macrocycle occurs. This fact produces a lowering of the melt temperature of BCL with respect to the free crystalline form. Figure 2b shows the thermograms of the BCL/SBE-β-CyD system. In this case, the thermogram of the BCL/SBE-β-CyD physical mixture also showed the superimposition of the endothermic events present in the free components, that is, a band at about 120 °C, due to the loss of crystalline water from the melting peak of the crystalline BCL, at 193 °C, and finally, the degradation of the macrocycle at a temperature of around 280 °C. A different trend characterizes the thermogram of the inclusion complex. In fact, the melting peak of the crystalline BCL is absent, while a low and broad endothermic signal is present at about 150 °C, probably showing the solid-solid BCL/SBE-β-CyD interaction.

**Figure 3:**
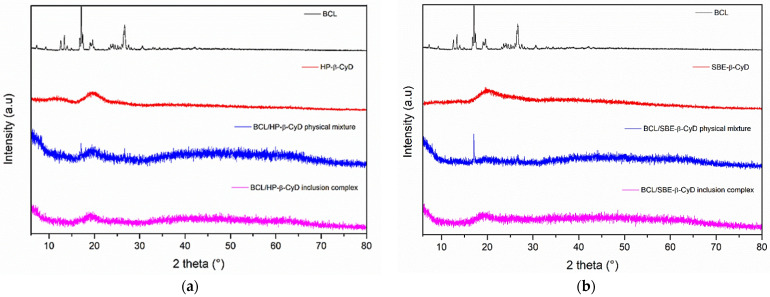
X-ray diffraction patterns of BCL/HP-β-CyD (**a**) and BCL/SBE-β-CyD (**b**) systems.

The amorphous nature of the freeze-dried complexes was confirmed by X-ray analysis. Figure 3a shows the X-ray diffractograms of free BCL, free HP-β-CyD, the BCL/HP-β-CyD inclusion complex, and the BCL/HP-β-CyD physical mixture in the same molar ratio of the complex. The X-ray diffraction pattern of BCL shows intense and sharp peaks at 2θ of 6.35, 12.29, 17.79, 23.25, 24.22, 29.1, 29.42, 31.55, and 34.62, evidencing the crystalline nature of the drug. HP-β-CyD showed no sharp peak but only a broad hump at 2θ of about 18, demonstrating its amorphous nature. The BCL/HP-β-CyD freeze-dried sample showed no peak corresponding to free BCL, which is still present in the X-Ray diffraction pattern of the physical mixture, demonstrating the existence of a new amorphous solid phase in which solid-solid interaction between the drug and the macrocycle is realized.

Similar results were obtained for BCL/SBE-β-CyD system and the obtained X-ray diffraction patterns are shown in Figure 3b. In this case, we also observed a loss of crystallinity and the absence of BCL characteristics peaks in the freeze-dried sample, confirming the existence of a new, solid phase with amorphous characteristics.

### 3.2. In-Solution Characterization

**Figure 4:**
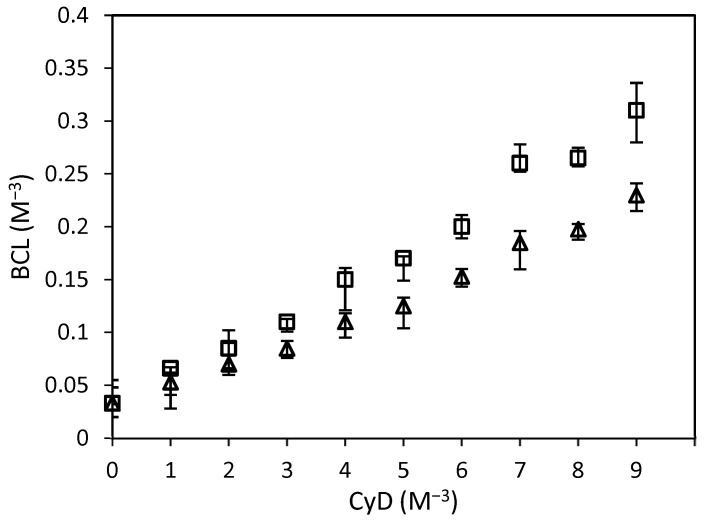
Phase-solubility diagrams of BCL/HP-β-CyD (squares) and BCL/SBE-β-CyD (triangles) complexes in PBS (pH 7.4) at 25.0 ± 0.1 °C.

Phase-solubility studies of BCL in the presence of increasing amounts of HP-β-CyD and SBE-β-CyD were carried out in water at 25.0 ± 0.1 °C [35]. Figure 4 shows the obtained isotherm. A linear increase in BCL solubility with increasing amounts of CyDs was observed. In both cases, the diagrams are of the A_L-_type, demonstrating the presence of soluble complexes within the used CyDs concentration range. The slope of the straight line was below 1 for both systems, proving the formation of complexes in the 1:1 molar ratio. The stability constant (K_c_) of the complexes, determined following the equation created by Higuchi and Connors [37], was about 9018 M^−1^ and 4530 M^−1^, for BCL/HP-β-CyD and BCL/SBE-β-CyD complexes, respectively, thereby confirming the higher affinity of HP-β-CyD with the drug than SBE-β-CyD. The higher Kc values observed for the two complexes compared to those of the BCL/β-CyD inclusion complex, as reported in the literature [32], were due to the presence of the hydroxypropyl and sulfobutylether groups, which extend the lipophilic cavity of the macrocycle, compared to native β-CyD, improving its affinity for the lipophilic drug [47]. On the other hand, the Kc value of the complexes described herein is higher than the complexes of BCL with methyl- and acetyl-β-Cyd, as described by other authors [35]. This trend could demonstrate a different interaction of BCL with the CyD substituents, one that is higher for the hydroxypropyl group than for the other substituents. This produces a high affinity of BCL with the cavity of HP-β-CyD.

At the maximum CyD concentration (9 mM), 10-fold and 7-fold increases in BCL solubility were observed in the presence of HP-β-CyD and SBE-β-CyD, respectively. There is a close correlation between the increase in solubility and the stability of the complexes; higher stability corresponds with the greater apparent solubility of the included BCL.

**Figure 5:**
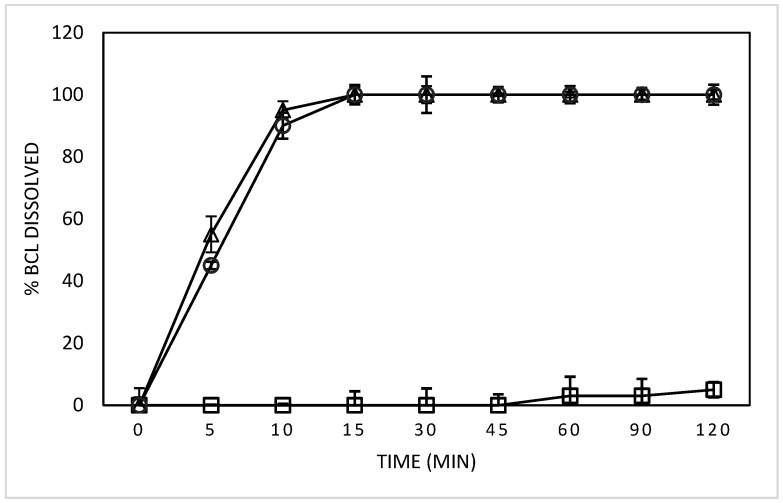
Dissolution profiles of the free and complexed BCL in PBS (pH 6.8) at 37 ± 0.5 °C, with free BCL (squares), BCL/HP-β-Cyd inclusion complex (triangles), and the BCL/SBE-β-CyD inclusion complex (circles).

As a result of the increased solubility of the complexed BCL and of the increased wettability of the solid complex, due to its amorphous state, the quantitative dissolution of both complexes was observed within 10 min (Figure 5).

The interaction of BCL with the CyDs was investigated in solution by UV-vis and ^1^H-NMR spectroscopy.

**Figure 6:**
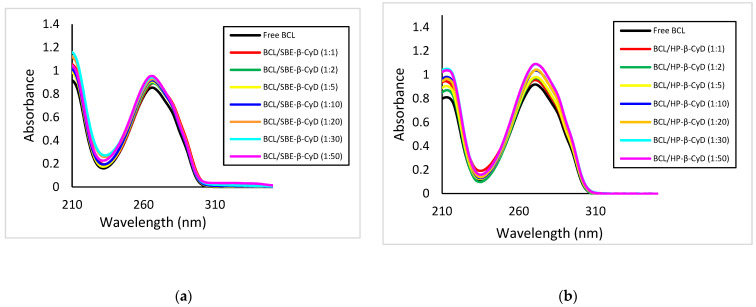
UV-vis spectra of BCL alone and in the presence of an increasing amount of SBE-β-CyD (**a**) and HP-β-CyD (**b**) in water/methanol solution (60/40, *v*/*v*).

BCL shows a UV band at 272 nm, due to the π→π* transition of the aromatic rings, which increases in intensity as the concentration of both CyDs increases (Figure 6a,b), particularly in the case of HP-β-CyD. This hyperchromic effect is the result of the variation of the local polarity produced by the inclusion of the BCL chromophore electrons within the apolar Cyd cavities, thereby passing from a polar environment (aqueous solution) to an apolar one (the internal CyD cavities). The higher hyperchromic effect produced by HP-β-CyD, compared to SBE-β-CyD, confirms the greater affinity of this CyD for the drug than the other macrocycle.

**Figure 7:**
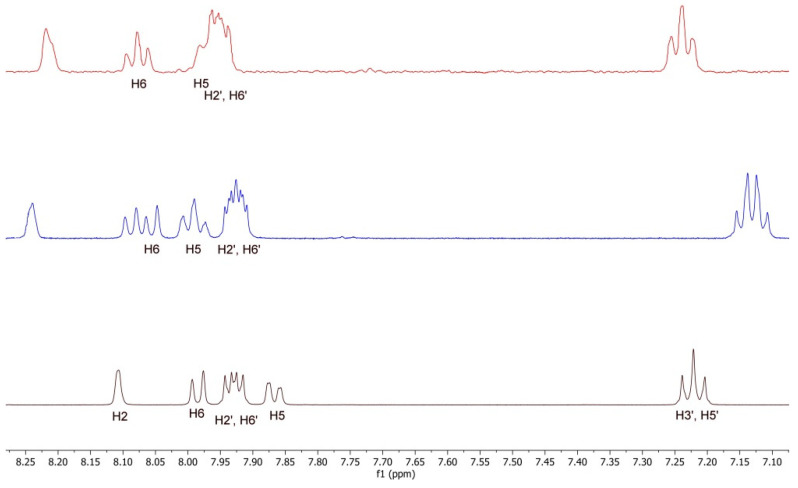
Stacked portions of the ^1^H-NMR spectra, relative to the free BCL (bottom), BCL/SBE-β-CyD (blue line), and BCL/HP-β-CyD (red line) inclusion complexes. Only those diagnostic signals relative to BCL are shown.

Among the spectroscopic techniques, nuclear magnetic resonance (NMR) has been used as a powerful tool for the study of inclusion complexes, since the chemical and electronic environments of the protons are modified by the interactions between the host and the guest molecules during the complexation; therefore, a chemically induced shift (CIS) of the corresponding protons has been observed. Unfortunately, the HP-β-CyD and SBE-β-CyD, as with most of the substituted CyDs, can be considered to be a statistical mixture of the different stereoisomers, with unresolved broad peaks, making it almost impossible to follow the chemical shifts of its H_3_ and H_5_ protons, although these were identified through 2D COSY spectra [48]. Therefore, the formation of the BCL/CyD inclusion complexes were deduced from the chemical shift changes observed in ^1^H NMR of the BCL aromatic protons, measured in the absence and presence of the two CyDs. The most interesting and evident variations of the chemical shift are in a range between 7.10 and 8.30 ppm, which was free of the broad and unsolved peaks of CyDs. Figure 7 shows the stacked portions of the ^1^H NMR spectra of BCL, together with the HP-β-CyD and SBE-β-CyD inclusion complexes; the observed chemical shifts are listed in Table 1.

Significant downfield shifts in the H_2_, H_5_, and H_6_ aromatic hydrogen atoms of BCL were observed for the two inclusion complexes. For the BCL/SBE-β-CyD inclusion complex, a positive Δδ of 0.18, 0.17, and 0.13 was observed for H_2_, H_5_, and H_6_, respectively, indicating that the 4-cyano-3-(trifluoromethyl)phenyl ring is encapsulated in the CyD cavity. Similarly, for the BCL/HP-*β*-CD inclusion complex, the Δδ values of the corresponding H_2_, H_5_, and H_6_ protons (Δδ 0.11, 0.10, and 0.10, respectively) were diagnostic for the same environmental conditions, due to the inclusion into CyD cavities.

### 3.3. Molecular Dynamics (MD) Studies

**Figure 8:**
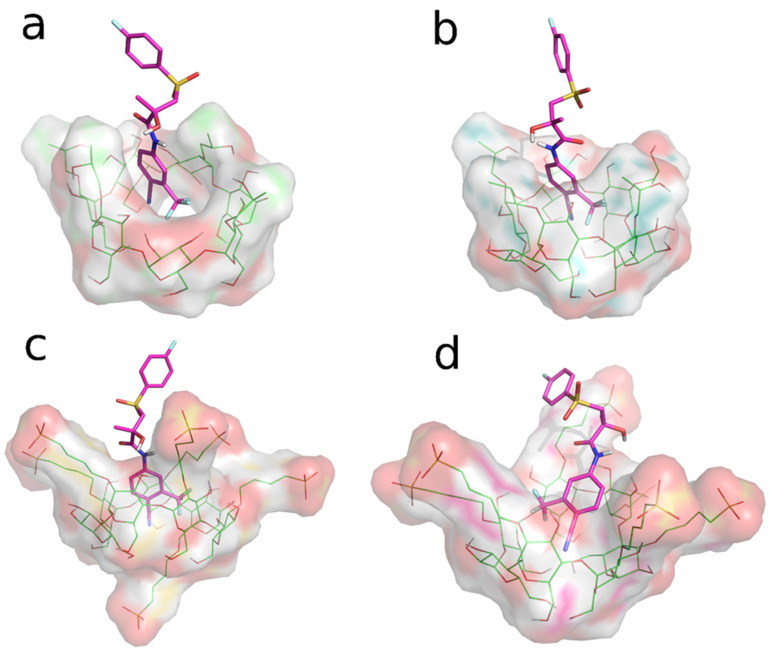
Representative structures of the most populated clusters from the REST MD simulations. The BCL enantiomers are represented by the magenta sticks, whereas CyDs are depicted in green sticks (carbon atoms). Non-carbon atoms are coloured according to the following standard scheme: S-Yellow, N-Blue, O-Red, F-Cyan. Inclusion complexes of: (**a**) *R*-BCL/HP-β-CyD; (**b**) *S*-BCL/HP-β-CyD; (**c**) *R*-BCL/SBE-β-CyD; (**d**) *S*-BCL/SBE-β-CyD.

**Figure 9:**
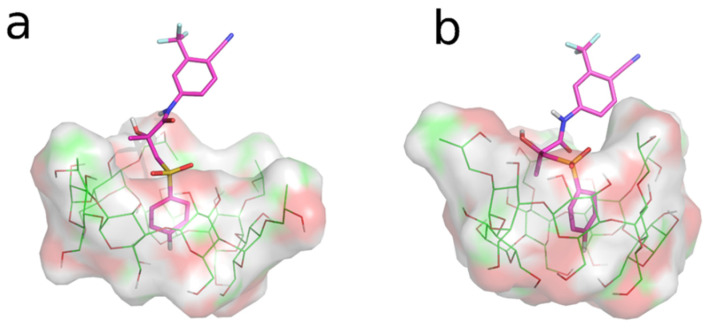
Representative structures of the second-rank clusters from the REST MD simulations. The BCL enantiomers are represented by magenta sticks, while HP-β-CyD is depicted by green sticks (carbon atoms). Non-carbon atoms are coloured according to the following standard scheme: S-Yellow, N-Blue, O-Red, F-Cyan. The inclusion complexes are of: (**a**) *R*-BCL/HP-β-CyD; (**b**) *S*-BCL/HP-β-CyD.

The REST MD simulations were used as an unbiased dynamic docking approach, with the aim of gaining insight into the interaction of the two BCL enantiomers (*R*-BCL and *S*-BCL) with HP-β-CyD and SBE-β-CyD. Trajectory snapshots from the REST MD replica 0 (t = 300 K) were clustered according to atomic RMSD values (see Section 2.10.2). Cluster analysis (given in Table 2) of the REST MD trajectories suggested that both the enantiomers of BCL preferentially bind the two CyDs by the inclusion of the 4-cyano-3-(trifluoromethyl)phenyl ring in the CyD cavities (Figure 8a,d); both CyDs seem to possess a higher affinity toward the eutomer (*R*-BCL). A single binding mode is observed for BCL/SBE-β-CyD interaction, while BCL appears capable of interacting with the HP-β-CyD cavity in different binding modes, although to a lesser extent. In particular, the second-ranking clusters of *R*-BCL/HP-β-CyD and *S*-BCL/HP-β-CyD interactions showed the inclusion of the fluorophenyl ring into the CyD cavity for both the enantiomers (Figure 9a,b).

### 3.4. In Vitro Anticancer Activity on PC3 and DU145 Cell Lines

The anticancer activity of BCL/HP-β-CyD and BCL/SBE-β-CyD inclusion complexes was assayed, compared to the free drug, on PC3 and DU145 human prostate cancer cell lines. Both PC3 and DU145 cells are particularly suitable for evaluating the pharmacological effects of new drugs or drugs delivered using innovative systems because they are highly aggressive in both in vitro and in vivo models. These two cell lines are generally reported as not expressing the androgen receptor (AR) [49,50]. However, some authors [51] have demonstrated the presence of detectable nuclear AR in both DU145 and PC3 cells.

**Figure 10:**
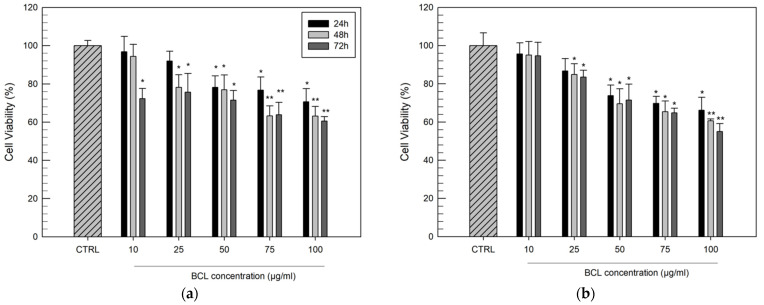
Cytotoxic effects of free BCL evaluated on DU-145 cells (**a**) and PC-3 cells (**b**), as a function of drug concentrations (10–100 µg/mL) and duration of exposure (24, 48, and 72 h). Results are expressed as the mean values of three different experiments ± standard deviation. * *p* < 0.05 and ** *p* < 0.001 of BCL concentration, compared with untreated cells (CTRL).

Firstly, we evaluated the suitable concentration of BCL that is able to inhibit the DU-145 and PC-3 cell lines proliferation with a certain potency. Since BCL is a poorly water-soluble drug, it was necessary to solubilize the drug using DMSO (a stock solution of 2 mg/mL); the results reported in Figure 10 were normalized considering the cytotoxic effects of DMSO alone, tested at the same concentration used to administer the BCL.

As can be seen, PC-3 cells seem to be more resistant to the antiproliferative effect of BCL, as reported by other authors [52]. In any case, a high concentration of BCL (100 µg/mL) and a long-timespan treatment (72 h) were necessary to record a reduction of cell viability of greater than 40% for both cell lines. These results confirmed previous studies that described BCL as a poorly effective drug regarding hormone-independent cell lines [52]. Starting from these preliminary in vitro results, we evaluated the influence of the BCL complexation within HP-β-CyD and SBE-β-CyD cavities on its in vitro anticancer activity by administering the drug to the cells at concentrations of 25, 50, and 75 µg/mL. The same concentration range had already been chosen by other research groups, which proposed other delivery systems [53,54].

**Figure 11:**
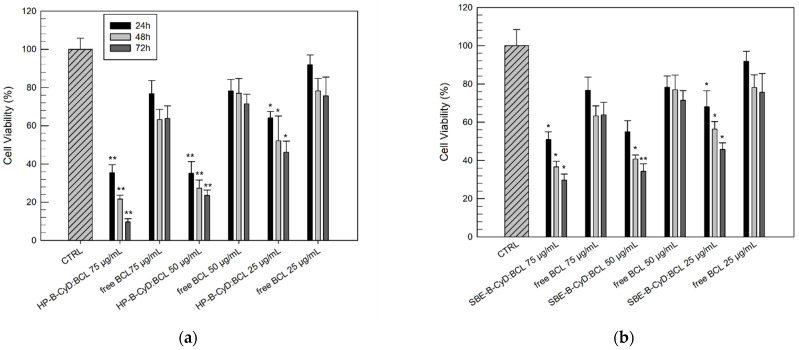
Cytotoxic effects of the BCL/HP-β-CyD (**a**) and BCL/SBE-β-CyD (**b**) inclusion complexes, in comparison to free BCL, on DU-145 cells as a function of drug concentrations (75, 50, and 25 µg/mL) and duration of exposure (24, 48, and 72 h). Results are expressed as the mean values of three different experiments ± standard deviation. * *p* < 0.05 and ** *p* < 0.001 of the complexed BCL concentration, compared with the same concentration of free BCL.

**Figure 12:**
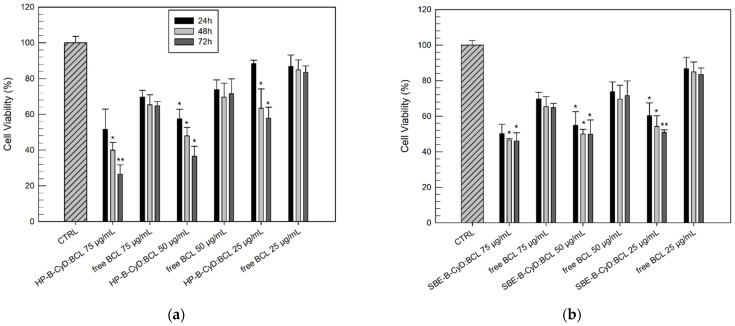
Cytotoxic effects of BCL/HP-β-CyD (**a**) and BCL/SBE-β-CyD (**b**) compared to the free BCL evaluated on PC-3 cells as a function of drug concentrations (75, 50, and 25 µg/mL) and duration of exposure (24, 48, and 72 h). Results are expressed as mean values of three different experiments ± standard deviation. * *p* < 0.05 and ** *p* < 0.001 of complexed BCL concentration, compared with the same concentration of free BCL.

The cytotoxic effects, also in this case, were evaluated as a function of the incubation time (24, 48, and 72 h) to define both the effective concentration and the effects of the duration of exposure. Figure 11 and Figure 12 show the results (in terms of cell viability percentage) of the MTT assay carried out on the DU-145 and PC3 cell lines, respectively, comparing the free drug and BCL/HP-β-CyD or BCL/SBE-β-CyD inclusion complexes.

Observing Figure 11a, the benefits, in terms of improved cytotoxic effects, of BCL when tested in the form of a BCL/HP-β-CyD inclusion complex on DU-145 are immediately evident. At 25 µg/mL and after 24 h, we already recorded significantly reduced cell viability (%) for the BCL/HP-β-CyD inclusion complex, compared to the free drug (64.11 ± 3.41% and 91.90 ± 5.14%, respectively). Moreover, the antiproliferative effects of the BCL/HP-β-CyD inclusion complex are more evident when compared with free BCL at a concentration equal to 75 µg/mL, after 72 h of treatment. In this case, the difference, in terms of cell viability, between the BCL/HP-β-CyD inclusion complex and the free drug was very evident (Figure 11a). The DU-145 cells treated with the inclusion complex showed a very low survival rate (9.61 ± 1.74%), compared to the cells treated with the same concentration of free BCL (63.82 ± 6.52%).

Similar results were obtained for the PC-3 cells treated with the BCL/HP-β-CyD inclusion complex (Figure 12). Despite the fact that the PC-3 cells showed more resistance to the antiproliferative activity of BCL, its complexation within the HP-β-CyD cavity produced a more cytotoxic effect on PC-3 cells compared to the free drug (Figure 12a). In detail, after 72 h of treatment, the lowest tested concentration of the BCL/HP-β-CyD inclusion complex (25 µg/mL) induced a significant reduction in cell viability with respect to the same concentration of the free drug (57.80 ± 6.16% and 83.50 ± 3.59% of cell viability, respectively). Moreover, using the lowest BCL/HP-β-CyD concentration (25 µg/mL), we recorded a similar cytotoxic effect to that obtained by using the free BCL at the highest concentration (100 µg/mL) (57.80 ± 6.16% and 54.99 ± 4.14% of the cell viability for BCL/HP-β-CyD and free BCL, respectively).

A similar trend was observed when cells were treated with the BCL/SBE-β-CyD inclusion complex, even if it was in a lower extension than the BCL/HP-β-CyD inclusion complex (see Figure 11b and Figure 12b).

The improved anticancer activity observed for BCL/HP-β-CyD and BCL/SBE- β-CyD inclusion complexes with respect to free BCL was due to the solubilizing effect exerted by the macrocycles on BCL. The cytotoxic effect of BCL in its free form was limited by its poor solubility. In fact, despite the solubilization of the drug in DMSO, successive dilution with the culture medium produced the partial precipitation of the drug; then, not all the assayed dose was available to interact with the cells. The BCL/HP-β-CyD and BCL/SBE- β-CyD inclusion complexes were totally solubilized in the culture medium; thus, free BCL, which was released by the complex as a function of its K_c_ value, was able to better interact with the target cells, showing greater anticancer activity. Furthermore, the ability of CyDs to interact with the membrane component, altering their permeability, must be considered [55,56] and could explain the greater efficacy of BCL/HP-β-CyD, compared to the BCL/SBE-β-CyD inclusion complex. In fact, as demonstrated by different authors, HP-β-CyD shows a higher influence on biomembranes than SBE-β-CyD [57,58].

## 4. Conclusions

In this work, we achieved soluble inclusion complexes of BCL with HP-β-CyD and SBE-β-CyD, intended for prostate cancer treatment. The DSC studies and X-ray diffraction analysis demonstrated the amorphous nature of the solid complexes obtained via the freeze-drying method. The complexes significantly improve the water solubility of BCL, permitting a fast and complete dissolution of the solids within 15 min. Both ^1^H-NMR and theoretical studies demonstrated the inclusion of the 4-cyano-3-(trifluoromethyl)phenyl ring within the CyD cavities. The complexation increased the antiproliferative effect of BCL significantly on DU145 and PC3 human cancer prostate cell lines, probably not only due to the increased water solubility of BCL but also due to the destabilizing effect exerted by the CyDs on cells, with a consequent increase in drug permeation.

The obtained results show the high potential of HP-β-CyD and SBE-β-CyD as complexing agents for BCL, permitting the realization of fast-dissolving formulations for oral administration. In this way, the oral bioavailability of BCL could be improved. Furthermore, due to the safety of HP-β-CyD and SBE-β-CyD, liquid formulations for parenteral administration can be prepared. In vivo studies are underway to confirm these results.

## Figures and Tables

**Table 1:** ^1^H NMR chemical shifts in δ and Δδ of the BCL protons in a free state and of BCL/HP-β-CyD and BCL/SBE-β-CyD complexes (1:1 molar ratio; 9 mM in D_2_O/MeOD (0.8/0.2 mL) solution). For multiplet, doublet, or AB systems, the reported δ is referred to as the centered signal.

Protons	BCL	BCL/HP-β-CyD	Δδ *	BCL/SBE-β-CyD	Δδ *
CH_3_	1.52 (s)	1.54	0.02	1.55	0.03
CH_2_	3.94 (AB system)	n.d.		n.d.	
H_3′,5′_	7.22 (t)	7.24	0.02	7.17	−0.05
H_5_	7.86 (dd)	7.96	0.10	8.03	0.17
H_2′,6′_	7.93 (m)	7.95	0.02	7.96	0.03
H_6_	7.98 (dd)	8.08	0.10	8.11	0.13
H_2_	8.11 (s)	8.22	0.11	8.29	0.18

* Δδ = δ_complex_ − δ_free._

**Table 2:** Clustering of the REST MD simulation trajectories. Clusters populated by fewer than 100 members were not considered.

CyD	Ligand	Cluster ^a^	Cluster Size
HP-β-CyD	*R*-BCL	1	1300
		2	732
		3	335
	*S*-BCL	1	785
		2	438
SBE-β-CyD	*R*-BCL	1	2181
	*S*-BCL	1	938

^a^ Ranking by cluster population.

## Data Availability

The data presented in this study are available on request from the corresponding author. The data are not publicly available due to privacy.
